# CAD/CAM implant surgical guides: maximum errors in implant positioning attributable to the properties of the metal sleeve/osteotomy drill combination

**DOI:** 10.1186/s40729-018-0146-2

**Published:** 2018-11-09

**Authors:** Dimitrios Apostolakis, Georgios Kourakis

**Affiliations:** Private Practice, Dental Radiology in Crete, Plateia 1866, No 39, 73100 Chania, Crete Greece

**Keywords:** CAD, CAM, Implant guide, 3d printing, Osteotomy, Stereolithography

## Abstract

**Background:**

The purpose of this study is to provide the relevant equations and the reference tables needed for calculating the maximum errors in implant positioning attributed to the properties of the mechanical parts of any CAD/CAM implant surgical guide, especially the in-office manufactured ones.

**Methods:**

An algorithm was developed and implemented in C programming language in order to accurately calculate the maximum error at the apex, error at the neck, vertical error at the apex and deviation of implant axis, between the planned and the actual implant position. The calculations were based on the parameters of total length (= implant length + offset), offset (distance from neck of implant to the lip of the metal sleeve), clearance (space between the bur and the sleeve), sleeve length. The variability of the parameters was constrained: (1) implant length, 8–18 mm; (2) sleeve length, 4–7 mm; (3) clearance, 50–410 μm; and (4) offset values, 6–17 mm. Multiple regression analysis was conducted to quantify the relationship between the error at the apex and the error at the neck and various predictors.

**Results:**

The equations used for the bespoke estimation of the errors in implant positioning along with three reference tables of the various errors tabulated are presented. The maximum error at the apex of the implant was computed 2.8 mm, the maximum deviation of the implant axis 5.9° and the maximum error at the neck (entrance) of the implant was estimated 1.5 mm. The vertical error between the planned and actual implant position can be considered negligible (< 0.1 mm).

**Conclusions:**

The results of this study compute part of the expected differences in final clinical implant position when any CAD/CAM surgical guide is used. Given that the implantologist, with the capability of an in-office digital designed and 3d printed surgical guide, can readily decide upon the dimensions of the metal sleeve, the clearance between the osteotomy bur and the sleeve, and the design of the guide in relation to the distance of the lip of the sleeve to the implant neck (offset), in order to minimise the inevitable errors.

## Background

Computer-aided designed and computer-aided manufactured (CAD/CAM) implant surgical guides are long recommended to reliably transfer a virtual treatment plan to the surgical field [1, 2]. The 3d-printed guide stands a basic part of a process commonly referred to as guided implant surgery (GIS) [3]. The outcome of this process has been shown to be relatively accurate [4, 5], even when the guide is in the hands of inexperienced surgeons [6].

The error in guided implant surgery, when defined as the difference between the planned and the actual position of the implant, is the cumulative result of flaws along the different stages of the procedure. Inaccuracies in the CBCT or CT acquisition process [7], the DICOM to STL conversion [8], the registration process of the different modes [3], the procedure followed for designing and manufacturing the surgical guide [9], the method used to stabilise the guide in the mouth (i.e. teeth, mucosa, bone) [10], the way the guide is manipulated by the surgeon and finally the quality and quantity and morphology of the local bone [4].

All of the current research concerning the mechanical parts of a CAD/CAM surgical guide has so far investigated the accuracy of implant guides designed and manufactured professionally by companies specialising in the field of medicine and dentistry [11–16]. These companies usually provide, in addition to the guide and software, their own correspondent surgical kit, especially designed to perform solely with their guide.

In-office 3d printing with low-cost fused deposition modelling (FDM) or desktop stereolithography apparatus (SLA) printers and freeware provides a cheaper alternative for manufacturing surgical guides with materials and components supplied from the free market. In-office 3d printing gives the opportunity to the implantologist to readily produce a CAD/CAM guide and place implants using the surgical kit at his current disposal. The production of such a 3d guide may pertain the same errors as a commercially constructed guide does, with the exception that now the implantologist is oblivious to the magnitude and, as a result, the clinical significances of these errors.

It is the aim of this paper to compute the maximum errors in the positioning of the implants with relation to the basic mechanical components of a 3d surgical guide/surgical kit combination taking into account the positional and dimensional properties of the guide’s metal part (sleeve) and the dimensional properties of any osteotomy bur used. The analytical equations for the bespoke computation of the errors for any conceivable combination of the relevant parameters will be provided. Reference tables will be reported facilitating the in-office production of any 3d surgical implant guide.

## Methods

**Fig. 1:**
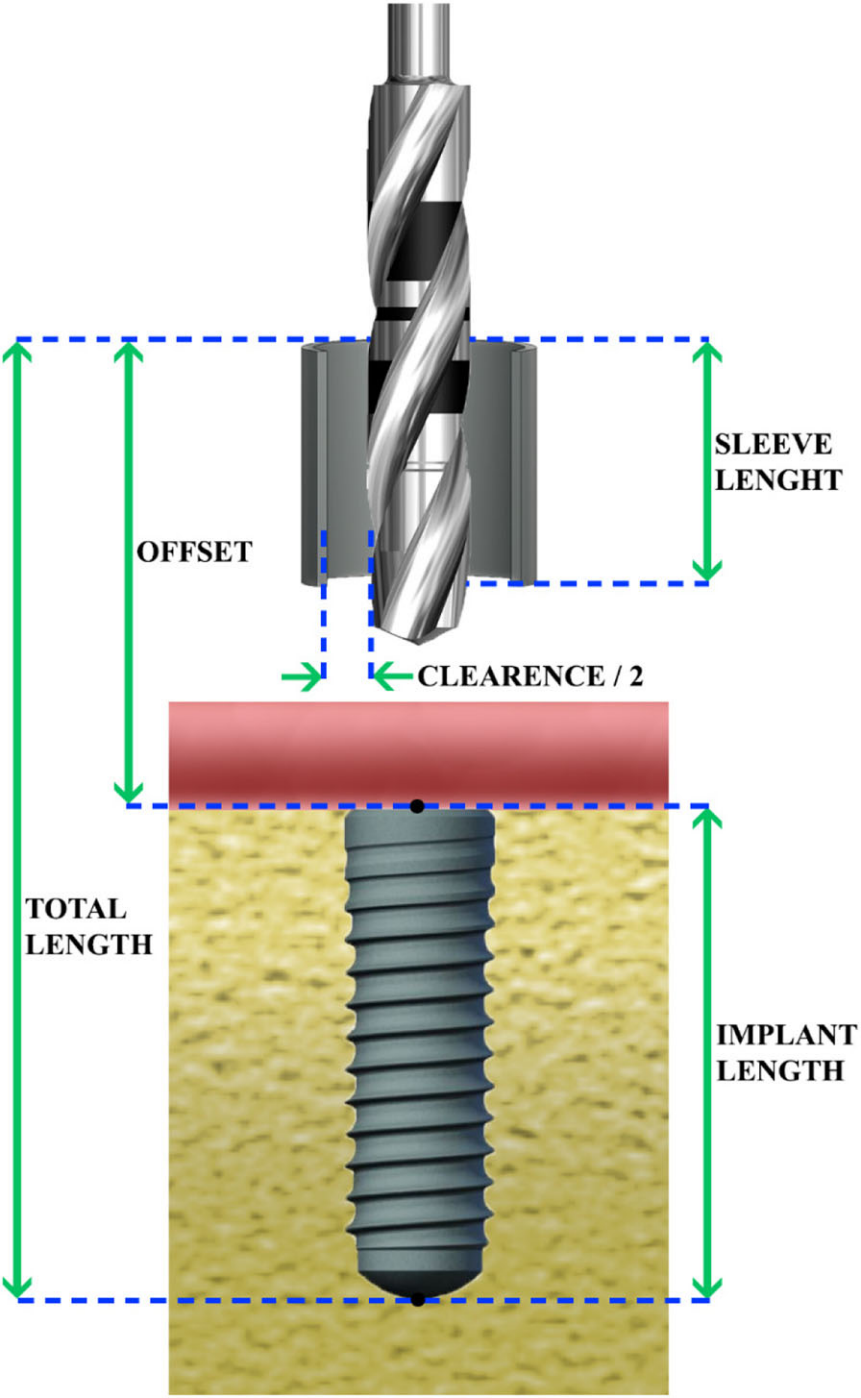
The parameters used for the calculation of the various errors and the deviation of implant axis

**Fig. 2:**
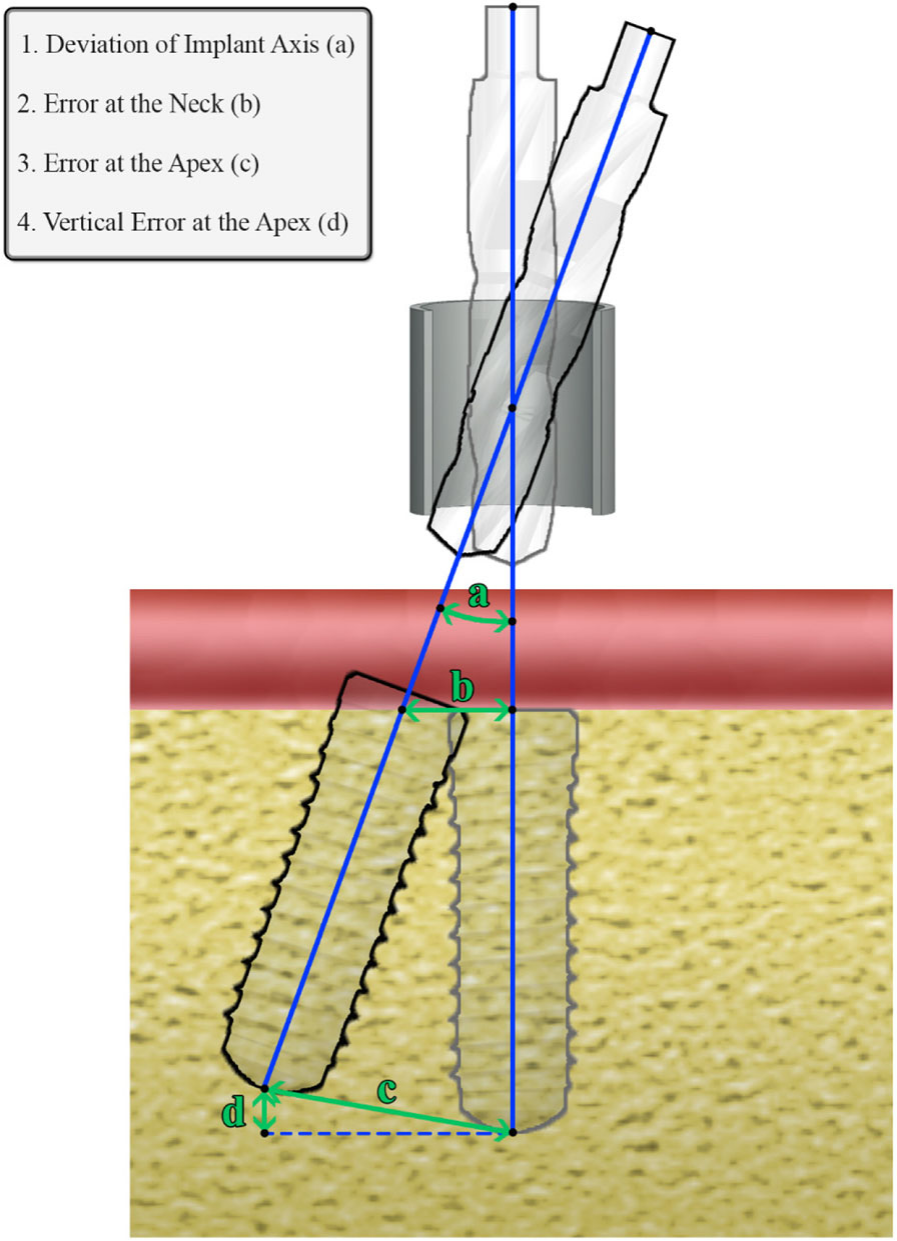
The various errors in implant positioning

For the estimation of the errors in implant positioning due to the properties of the metal sleeve/osteotomy drill combination, four [4] parameters are necessary: (1) sleeve length, (2) clearance (space between the bur and the sleeve), (3) implant length, and (4) offset (distance of the lip of the metal sleeve to the neck of the implant) (Figs. 1 and 2).

### Definitions

1. Basic size: the nominal size of the metal sleeve and the osteotomy drill given by the manufacturer.
2. Sleeve length: the total length of the metal sleeve inserted into the surgical guide, including any lip protruding on the occlusal surface of the guide.
3. Offset: the distance between the neck of the implant and the occlusal surface of the lip of the metal sleeve.
4. Clearance: the difference between the inner diameter of the metal sleeve and the diameter of the osteotomy bur.
5. Implant length: the nominal length of the implant from the neck to the apex.
6. Total length: the sum of implant length and offset.
7. Error at the apex: the Euclidian distance between the apex of the implant at the planned position and the apex at the more extreme position permissible by the sleeve/drill combination (measured from the central implant axis).
8. Error at the neck: the Euclidian distance between the neck of the implant at the planned position and the neck of the implant at the more extreme position permissible by the sleeve/drill combination (measured from the central implant axis).
9. Deviation: the angle in degrees between the central axis of the implant on the planned position and the same axis on the most extreme permissible position.
10. Vertical error at the apex: the distance of the final position of the apex of the implant to the horizontal plane where the point of the planned position of the implant is included.

Based on the geometric analysis of the problem in hand, an algorithm was developed and implemented in C programming language. The purpose of this program was to readily and accurately compute the following maximum positioning errors, permissible by the different sleeve/drill/guide properties (Fig. 2):

1. Deviation of the implant axis in degrees,

2. Error at the neck in mm,

2. Error at the apex in mm,

4. Vertical error at the apex in mm.

These computations were based on the four parameters: (1) implant length, (2) sleeve length, (3) clearance and (4) offset (Fig. 1), and the results were tabulated in reference tables. The implant length and the offset were added together to form a new variable termed total length used exclusively on the calculations related to the error at the apex.

The variability of the parameters was constrained to reflect probable clinical conditions: (1) implant length between 8 and 18 mm, in 1 mm steps; (2) sleeve length between 4 and 7 mm, in 1 mm steps; (3) clearance between 50 and 410 μm, in 30 μm steps; and (4) offset values between 6 and 17 mm, in 1 mm steps. The minimum distance between the bottom of the metal sleeve (towards the bone) and the neck of the implant was set at the considered clinically appropriate distance of 2 mm.

Multiple regression was employed twice, with three independent variables each time to separate the effects of clearance, total length, sleeve length and offset on the values of the error at the apex and the error at the neck.

Microsoft® Excel 2016 32 bit was used for the statistical analysis. Significance level was set to *p* < 0.05.

All the values in the reference tables were rounded to one significant digit after the decimal point.

## Results

The range of the various maximum permissible errors due to the metal sleeve/osteotomy drill combination is presented in Table 1.

**Table 1:** Range of various maximum permissible errors as calculated in the present study

	Axis deviation (°)	Error at the neck (mm)	Error at the apex (mm)	Vertical error at the apex (mm)
Min	0.4	0.1	0.1	0.0
Max	5.9	1.5	2.8	0.1

Concerning the error at the apex, two reference tables were reported (Tables 2 and 3). In these tables, the deviation of the implant axis was also tabulated. A separate table (Table 4) tabulated the error at the neck.

**Table 2:** Error at the apex (mm) and deviation of implant axis (°) for sleeve lengths 4 and 5 mm

Sleeve length (mm)	Clearance (μm)	Deviation (degrees)	Total length (mm)															
			14	15	16	17	18	19	20	21	22	23	24	25	26	27	28	29
4.00	50.00	0.72	0.2	0.2	0.2	0.2	0.2	0.2	0.2	0.2	0.3	0.3	0.3	0.3	0.3	0.3	0.3	0.3
	80.00	1.15	0.2	0.3	0.3	0.3	0.3	0.3	0.4	0.4	0.4	0.4	0.4	0.5	0.5	0.5	0.5	0.5
	110.00	1.58	0.3	0.4	0.4	0.4	0.4	0.5	0.5	0.5	0.6	0.6	0.6	0.6	0.7	0.7	0.7	0.7
	140.00	2.00	0.4	0.5	0.5	0.5	0.6	0.6	0.6	0.7	0.7	0.7	0.8	0.8	0.8	0.9	0.9	0.9
	170.00	2.43	0.5	0.6	0.6	0.6	0.7	0.7	0.8	0.8	0.9	0.9	0.9	1.0	1.0	1.1	1.1	1.1
	200.00	2.86	0.6	0.7	0.7	0.8	0.8	0.9	0.9	1.0	1.0	1.1	1.1	1.2	1.2	1.3	1.3	1.4
	230.00	3.29	0.7	0.7	0.8	0.9	0.9	1.0	1.0	1.1	1.2	1.2	1.3	1.3	1.4	1.4	1.5	1.6
	260.00	3.72	0.8	0.8	0.9	1.0	1.0	1.1	1.2	1.2	1.3	1.4	1.4	1.5	1.6	1.6	1.7	1.8
	290.00	4.15	0.9	0.9	1.0	1.1	1.2	1.2	1.3	1.4	1.5	1.5	1.6	1.7	1.7	1.8	1.9	2.0
	320.00	4.57	1.0	1.0	1.1	1.2	1.3	1.4	1.4	1.5	1.6	1.7	1.8	1.8	1.9	2.0	2.1	2.2
	350.00	5.00	1.1	1.1	1.2	1.3	1.4	1.5	1.6	1.7	1.8	1.8	1.9	2.0	2.1	2.2	2.3	2.4
	380.00	5.43	1.1	1.2	1.3	1.4	1.5	1.6	1.7	1.8	1.9	2.0	2.1	2.2	2.3	2.4	2.5	2.6
	410.00	5.85	1.2	1.3	1.4	1.5	1.6	1.7	1.8	1.9	2.1	2.2	2.3	2.4	2.5	2.6	2.7	2.8
5.00	50.00	0.57		0.1	0.1	0.1	0.2	0.2	0.2	0.2	0.2	0.2	0.2	0.2	0.2	0.2	0.3	0.3
	80.00	0.92		0.2	0.2	0.2	0.2	0.3	0.3	0.3	0.3	0.3	0.3	0.4	0.4	0.4	0.4	0.4
	110.00	1.26		0.3	0.3	0.3	0.3	0.4	0.4	0.4	0.4	0.5	0.5	0.5	0.5	0.5	0.6	0.6
	140.00	1.60		0.4	0.4	0.4	0.4	0.5	0.5	0.5	0.5	0.6	0.6	0.6	0.7	0.7	0.7	0.7
	170.00	1.95		0.4	0.5	0.5	0.5	0.6	0.6	0.6	0.7	0.7	0.7	0.8	0.8	0.8	0.9	0.9
	200.00	2.29		0.5	0.5	0.6	0.6	0.7	0.7	0.7	0.8	0.8	0.9	0.9	0.9	1.0	1.0	1.1
	230.00	2.63		0.6	0.6	0.7	0.7	0.8	0.8	0.9	0.9	0.9	1.0	1.0	1.1	1.1	1.2	1.2
	260.00	2.98		0.7	0.7	0.8	0.8	0.9	0.9	1.0	1.0	1.1	1.1	1.2	1.2	1.3	1.3	1.4
	290.00	3.32		0.7	0.8	0.8	0.9	1.0	1.0	1.1	1.1	1.2	1.2	1.3	1.4	1.4	1.5	1.5
	320.00	3.66		0.8	0.9	0.9	1.0	1.1	1.1	1.2	1.2	1.3	1.4	1.4	1.5	1.6	1.6	1.7
	350.00	4.00		0.9	0.9	1.0	1.1	1.2	1.2	1.3	1.4	1.4	1.5	1.6	1.6	1.7	1.8	1.9
	380.00	4.35		1.0	1.0	1.1	1.2	1.3	1.3	1.4	1.5	1.6	1.6	1.7	1.8	1.9	1.9	2.0
	410.00	4.69		1.0	1.1	1.2	1.3	1.4	1.4	1.5	1.6	1.7	1.8	1.8	1.9	2.0	2.1	2.2

**Table 3:** Error at the apex (mm) and deviation of implant axis (degrees) for sleeve lengths 6 and 7 mm

Sleeve length (mm)	Clearance (μm)	Deviation (°)	Total length (mm)													
			16	17	18	19	20	21	22	23	24	25	26	27	28	29
6	50	0.5	0.1	0.1	0.1	0.1	0.1	0.2	0.2	0.2	0.2	0.2	0.2	0.2	0.2	0.2
	80	0.8	0.2	0.2	0.2	0.2	0.2	0.2	0.3	0.3	0.3	0.3	0.3	0.3	0.3	0.3
	110	1.1	0.2	0.3	0.3	0.3	0.3	0.3	0.3	0.4	0.4	0.4	0.4	0.4	0.5	0.5
	140	1.3	0.3	0.3	0.4	0.4	0.4	0.4	0.4	0.5	0.5	0.5	0.5	0.6	0.6	0.6
	170	1.6	0.4	0.4	0.4	0.5	0.5	0.5	0.5	0.6	0.6	0.6	0.7	0.7	0.7	0.7
	200	1.9	0.4	0.5	0.5	0.5	0.6	0.6	0.6	0.7	0.7	0.7	0.8	0.8	0.8	0.9
	230	2.2	0.5	0.5	0.6	0.6	0.7	0.7	0.7	0.8	0.8	0.8	0.9	0.9	1.0	1.0
	260	2.5	0.6	0.6	0.7	0.7	0.7	0.8	0.8	0.9	0.9	1.0	1.0	1.0	1.1	1.1
	290	2.8	0.6	0.7	0.7	0.8	0.8	0.9	0.9	1.0	1.0	1.1	1.1	1.2	1.2	1.3
	320	3.1	0.7	0.7	0.8	0.9	0.9	1.0	1.0	1.1	1.1	1.2	1.2	1.3	1.3	1.4
	350	3.3	0.8	0.8	0.9	0.9	1.0	1.1	1.1	1.2	1.2	1.3	1.3	1.4	1.5	1.5
	380	3.6	0.8	0.9	1.0	1.0	1.1	1.1	1.2	1.3	1.3	1.4	1.5	1.5	1.6	1.6
	410	3.9	0.9	1.0	1.0	1.1	1.2	1.2	1.3	1.4	1.4	1.5	1.6	1.6	1.7	1.8
7	50	0.4		0.1	0.1	0.1	0.1	0.1	0.1	0.1	0.1	0.2	0.2	0.2	0.2	0.2
	80	0.7		0.2	0.2	0.2	0.2	0.2	0.2	0.2	0.2	0.2	0.3	0.3	0.3	0.3
	110	0.9		0.2	0.2	0.2	0.3	0.3	0.3	0.3	0.3	0.3	0.4	0.4	0.4	0.4
	140	1.1		0.3	0.3	0.3	0.3	0.4	0.4	0.4	0.4	0.4	0.5	0.5	0.5	0.5
	170	1.4		0.3	0.4	0.4	0.4	0.4	0.4	0.5	0.5	0.5	0.5	0.6	0.6	0.6
	200	1.6		0.4	0.4	0.4	0.5	0.5	0.5	0.6	0.6	0.6	0.6	0.7	0.7	0.7
	230	1.9		0.4	0.5	0.5	0.5	0.6	0.6	0.6	0.7	0.7	0.7	0.8	0.8	0.8
	260	2.1		0.5	0.5	0.6	0.6	0.7	0.7	0.7	0.8	0.8	0.8	0.9	0.9	0.9
	290	2.4		0.6	0.6	0.6	0.7	0.7	0.8	0.8	0.8	0.9	0.9	1.0	1.0	1.1
	320	2.6		0.6	0.7	0.7	0.8	0.8	0.8	0.9	0.9	1.0	1.0	1.1	1.1	1.2
	350	2.9		0.7	0.7	0.8	0.8	0.9	0.9	1.0	1.0	1.1	1.1	1.2	1.2	1.3
	380	3.1		0.7	0.8	0.8	0.9	1.0	1.0	1.1	1.1	1.2	1.2	1.3	1.3	1.4
	410	3.4		0.8	0.8	0.9	1.0	1.0	1.1	1.1	1.2	1.3	1.3	1.4	1.4	1.5

**Table 4:** Error at the neck (mm)

Sleeve length (mm)	Clearance (μm)	Offset (mm)											
		6	7	8	9	10	11	12	13	14	15	16	17
4	50	0.1	0.1	0.1	0.1	0.1	0.1	0.1	0.1	0.2	0.2	0.2	0.2
	80	0.1	0.1	0.1	0.1	0.2	0.2	0.2	0.2	0.2	0.3	0.3	0.3
	110	0.1	0.1	0.2	0.2	0.2	0.2	0.3	0.3	0.3	0.4	0.4	0.4
	140	0.1	0.2	0.2	0.2	0.3	0.3	0.4	0.4	0.4	0.5	0.5	0.5
	170	0.2	0.2	0.3	0.3	0.3	0.4	0.4	0.5	0.5	0.6	0.6	0.6
	200	0.2	0.3	0.3	0.4	0.4	0.5	0.5	0.6	0.6	0.7	0.7	0.8
	230	0.2	0.3	0.3	0.4	0.5	0.5	0.6	0.6	0.7	0.7	0.8	0.9
	260	0.3	0.3	0.4	0.5	0.5	0.6	0.7	0.7	0.8	0.8	0.9	1.0
	290	0.3	0.4	0.4	0.5	0.6	0.7	0.7	0.8	0.9	0.9	1.0	1.1
	320	0.3	0.4	0.5	0.6	0.6	0.7	0.8	0.9	1.0	1.0	1.1	1.2
	350	0.4	0.4	0.5	0.6	0.7	0.8	0.9	1.0	1.1	1.1	1.2	1.3
	380	0.4	0.5	0.6	0.7	0.8	0.9	1.0	1.0	1.1	1.2	1.3	1.4
	410	0.4	0.5	0.6	0.7	0.8	0.9	1.0	1.1	1.2	1.3	1.4	1.5
5	50		0.0	0.1	0.1	0.1	0.1	0.1	0.1	0.1	0.1	0.1	0.1
	80		0.0	0.1	0.1	0.1	0.1	0.2	0.2	0.2	0.2	0.2	0.2
	110		0.1	0.1	0.1	0.2	0.2	0.2	0.2	0.3	0.3	0.3	0.3
	140		0.1	0.2	0.2	0.2	0.2	0.3	0.3	0.3	0.4	0.4	0.4
	170		0.2	0.2	0.2	0.3	0.3	0.3	0.4	0.4	0.4	0.5	0.5
	200		0.2	0.2	0.3	0.3	0.3	0.4	0.4	0.5	0.5	0.5	0.6
	230		0.2	0.3	0.3	0.3	0.4	0.4	0.5	0.5	0.6	0.6	0.7
	260		0.2	0.3	0.3	0.4	0.4	0.5	0.5	0.6	0.7	0.7	0.8
	290		0.3	0.3	0.4	0.4	0.5	0.6	0.6	0.7	0.7	0.8	0.8
	320		0.3	0.4	0.4	0.5	0.5	0.6	0.7	0.7	0.8	0.9	0.9
	350		0.3	0.4	0.5	0.5	0.6	0.7	0.7	0.8	0.9	0.9	1.0
	380		0.3	0.4	0.5	0.6	0.6	0.7	0.8	0.9	1.0	1.0	1.1
	410		0.4	0.5	0.5	0.6	0.7	0.8	0.9	0.9	1.0	1.1	1.2
6	50			0.0	0.1	0.1	0.1	0.1	0.1	0.1	0.1	0.1	0.1
	80			0.0	0.1	0.1	0.1	0.1	0.1	0.1	0.2	0.2	0.2
	110			0.1	0.1	0.1	0.1	0.2	0.2	0.2	0.2	0.2	0.3
	140			0.1	0.1	0.2	0.2	0.2	0.2	0.3	0.3	0.3	0.3
	170			0.1	0.2	0.2	0.2	0.3	0.3	0.3	0.3	0.4	0.4
	200			0.2	0.2	0.2	0.3	0.3	0.3	0.4	0.4	0.4	0.5
	230			0.2	0.2	0.3	0.3	0.3	0.4	0.4	0.5	0.5	0.5
	260			0.2	0.3	0.3	0.3	0.4	0.4	0.5	0.5	0.6	0.6
	290			0.2	0.3	0.3	0.4	0.4	0.5	0.5	0.6	0.6	0.7
	320			0.3	0.3	0.4	0.4	0.5	0.5	0.6	0.6	0.7	0.7
	350			0.3	0.4	0.4	0.5	0.5	0.6	0.6	0.7	0.8	0.8
	380			0.3	0.4	0.4	0.5	0.6	0.6	0.7	0.8	0.8	0.9
	410			0.3	0.4	0.5	0.5	0.6	0.7	0.8	0.8	0.9	1.0
7	50				0.0	0.0	0.1	0.1	0.1	0.1	0.1	0.1	0.1
	80				0.0	0.1	0.1	0.1	0.1	0.1	0.1	0.1	0.2
	110				0.1	0.1	0.1	0.1	0.1	0.2	0.2	0.2	0.2
	140				0.1	0.1	0.2	0.2	0.2	0.2	0.2	0.3	0.3
	170				0.1	0.2	0.2	0.2	0.2	0.3	0.3	0.3	0.3
	200				0.2	0.2	0.2	0.2	0.3	0.3	0.3	0.4	0.4
	230				0.2	0.2	0.2	0.3	0.3	0.3	0.4	0.4	0.4
	260				0.2	0.2	0.3	0.3	0.4	0.4	0.4	0.5	0.5
	290				0.2	0.3	0.3	0.4	0.4	0.4	0.5	0.5	0.6
	320				0.3	0.3	0.3	0.4	0.4	0.5	0.5	0.6	0.6
	350				0.3	0.3	0.4	0.4	0.5	0.5	0.6	0.6	0.7
	380				0.3	0.4	0.4	0.5	0.5	0.6	0.6	0.7	0.7
	410				0.3	0.4	0.4	0.5	0.6	0.6	0.7	0.7	0.8

Multiple regression analysis was conducted to examine the relationship between the error at the apex, the error at the neck and the various predictors. Table 5 summarises the analysis results. The multiple regression model for the error at the apex with all three predictors (total length, sleeve length, clearance) produced *R*^2^ = 0.98, *F* (3, 751) = 12,754, *p* < 0.0005. The multiple regression model for the error at the neck with all three predictors (offset, clearance, sleeve length) produced *R*^2^ = 0.97, *F* (3, 543) = 5677, *p* < 0.0005.

**Table 5:** Multiple regression coefficients (*p* < 0.0005)

	Sleeve length	Clearance	Total length	Offset
Error at the apex	− 0.1854	0.0037	0.0453	
Error at the neck	− 0.1041	0.0018		0.0461

The models:

$$ \mathrm{Error}\ \mathrm{at}\ \mathrm{the}\ \mathrm{apex}\ \left(\mathrm{mm}\right)=0.046\ast \left(\mathrm{total}\ \mathrm{length}\right)+0.0038\ast \left(\mathrm{clearance}\right)-0.19\ast \left(\mathrm{sleeve}\ \mathrm{length}\right) $$

$$ \mathrm{Error}\ \mathrm{at}\ \mathrm{the}\ \mathrm{neck}\ \left(\mathrm{mm}\right)=0.046\ast \left(\mathrm{offset}\right)+0.0018\ast \left(\mathrm{clearance}\right)-0.10\ast \left(\mathrm{sleeve}\ \mathrm{length}\right) $$

The values of total length and offset are in mm whilst the values of clearance are in μm.

As it is shown in Table 5, the total length, clearance and offset have positive and significant regression weights indicating that guided surgery with higher scores on these scales is expected to have higher errors in the implant positioning after controlling for the other variables in the model. The sleeve length has a significant negative weight indicating that after accounting for the rest of the variables, surgical guides with increased sleeve length are expected to have lower errors in implant positioning.

## Discussion

The purpose of a computer designed and computer manufactured (CAD/CAM) surgical guide is to provide the means for an accurate and reliable transfer of the computer-realised virtual treatment plan to the actual surgical field. The availability of the CBCT imaging modality should have led to an explosion of the usage of these guides, since they have been shown to be more accurate than freehand placement [17] and offer, in addition to the possibility of flapless surgery, the opportunity for a truly prosthetically driven implant placement [18]. However, increased costs, related to software acquisition and guide manufacturing, along with the special instrumentation required in most of the commercially available systems, in combination with an increased lead time between the completion of the virtual treatment plan and the delivery of the actual guide, make a lot of implantologists reluctant to the use of this technique.

Low-cost technologies for the in-office production of 3d guides already exist (SLA and FDM). A low-cost SLA 3d printer is advertised as the ideal for a dental practice with the same printer being tested for training in medical procedures [19]. Freeware for the design and the low-cost export of an STL file of the guide is available. The metal sleeves that are usually incorporated into the guide in order to accommodate the drill are offered in large numbers of internal and external dimensions. The dentist, almost by definition, possesses a high level of engineering and material skills [20]. He/she is currently able to accommodate their surgical need with a low-cost, fast-produced and fully CAD/CAM surgical guide. In the present study, we focus on the errors made during the manufacturing of such a guide and especially those depending on the way the metal components of the guide interrelate with the osteotomy bur and with the planned position of the implant. However, it should be stressed that the results of this study remain relevant for every implant surgical guide irrespective of the way it is manufactured, 3d-printed or other.

Computer-aided implant surgery is not flawless, even when the guides are manufactured by experts in 3d printing. Three recent systematic reviews have demonstrated errors between the planned and the final implant position. In the study of Schneider et al. [5], the review revealed a mean deviation of 1.07 mm (95% CI 0.76–1.22 mm) at the entry point, 1.6 mm (95% CI 1.26–2 mm) at the apex and a mean angular deviation of the implant axis of 5.3° (95% CI 3.94–6.581). In the study of Van Assche et al. [21], the mean error at the entry point was 0.99 mm (range 0–6.5 mm), at the apex 1.2 mm (range 0–6.9 mm) and the mean axis deviation was 3.81° (range 0–24.9 degrees). In the study of Tahmaseb et al. [17], the mean error at the entry point was 1.12 mm (maximum 4.5 mm) and 1.39 mm at the apex (maximum 7.1 mm). These reported errors are the summation of flaws in every stage of the procedure in the guided implant surgery: scanning, processing, manufacturing, surgery and not exclusively because of the properties of the mechanical parts of the guide.

A relatively small number of studies are concerned with the errors in the implant positioning due to the mechanical components of the guides. All of them refer to commercially available systems and try to evaluate or even improve these existing systems, elaborating in the mechanical errors of the particular system used [11–16].

Our study is free from the constraints of commercially available systems, but our equations and tables can be used to estimate errors generated by these systems when certain parameters are known. Our study calculates the maximum errors expected by the mechanical components of the computerised implant surgery process, with the understanding that the implantologist involved in the in-office printing has to have a knowledge of the dimensions, the design and the tolerances of the components he will use for the manufacturing of the guide. Cassetta et al. [13] estimated that 62.7% of the total implant positioning error was due to the properties of the sleeve/ drill combination, when the Materialise Safe® guide system was used. Even though it is recommended that the osteotomy should be performed without exerting force to the guide [12] and with the bur led parallel to the long axis of the sleeve, this is not possible in a number of cases, especially where the mouth opening is a limiting factor or when an oblique bone ridge is encountered. It is, therefore, probable that during surgery, the drill is tilted inside the sleeve, changing the final position of the implant. Then the metallic components with their dimensions and tolerances define the maximum permissible errors. Obviously, if the drill rotates exactly at the centre of the sleeve, no error is expected. It should be noted that when a sleeve/key/drill combination is used, the expected errors of the mechanical parts should be estimated taking into account the bigger clearance value created by the key usage. To our knowledge, this is the first study to comprehensibly calculate the errors by taking into consideration the parameters that contribute to the 62.7% of the total error in guided implant surgery, as Cassetta et al. [13] stated.

We provide the analytical equations that give the opportunity to the implantologist to calculate the errors of interest for every conceivable situation, even for cases not tabulated by us. Using our simple models, the surgeon is provided with unique information about a large part of the probable errors expected in guided surgery and he/she can include these values in a risk assessment model for the results of the implant surgery.

Elaborating further on the equations, it can be seen that for every 1 mm of increase in total length (implant length and/or offset), the errors increase by 46 μm. For every 50 μm increase in clearance, our models predict an increase in the error at the apex of 190 μm and in the error at the neck of 90 μm. Finally, for every 1 mm increase in the sleeve length, we anticipate a decrease in the error of implant positioning in the apex of 190 μm and in the error at the position of the implant neck of 100 μm. The Deviation of the implant axis is exclusively dependent on the sleeve length and the clearance and can easily be calculated. We found, in tantum with other studies, and as expected by the mathematical properties of the computation, that a longer implant with a short metal sleeve away from the neck of the implant (large offset) and a sleeve/bur combination with a large clearance will result in a large error at the neck of the implant, a larger error at the apex and a large deviation angle of the implant axis.

As an example on the implementation of our equations, we could simulate the (pilot) osteotomy for the positioning of an implant with a length of 12 mm. The implant will be placed with a 2.8 mm of diameter and 20.4 mm of length (with 0.4 mm tip), bur. The metal sleeve will be of 5 mm in length, including the lip of the sleeve and with an internal diameter of 2.89 mm. As a result, the clearance will be 90 μm. The offset is estimated as 8 mm and the total length at 20 mm, since 0.4 mm of the length of the bur is its tip and we do not expect the implant to reach that depth (The osteotomy hole is usually longer than the implant length but the error is calculated by the actual implant length). Under these parameters (total length = 12 + 8 = 20 mm, sleeve length = 5 mm, clearance = 90 μm), we calculate for the maximum errors expected due to the sleeve/drill combination:

Error at the apex = 0.29 mm

Error at the neck = 0.03 mm

And the maximum error of the implant axis deviation according to Table 4 is about 1.26°.

In addition to the equations, we produced in total three tables to present the error at the apex, the error at the neck and the axis deviation for different combinations of parameters (Tables 2, 3 and 4). We tabulated the expected errors at the apex of the implants taking into account the total length (= implant length + offset). That way, we kept our tables as slim and readable as possible. The deviation of the implant axis, in degrees, is also shown in the same table.

The vertical error at the apex of the implant was not tabulated in our study. It was estimated that the maximum vertical error at the apex was not probable to exceed 0.1 mm and therefore most of the cells on the table would be of zero value, after rounding. However, the small theoretical vertical error is in contradiction with actual final implant positions. Lee et al. [16] reported a mean vertical error of 0.935 ± 0.376 mm whilst Vercruyssen et al. [22] reported a mean overall vertical error of 0.9 ± 0.8 mm, concluding that this vertical error was the largest of all the errors possible. It seems that the vertical error is not due to properties of the mechanical components. Other factors such as the vertical sitting of the guide or the roughness of the 3d-printed sleeve may contribute as well. It is of importance that in most systems the length of the osteotomy should be longer than the length of the implant. In the case of in-office 3d printing, this has to be taken into consideration because a virtual osteotomy with the nominal length of the implant will lead to a more clinically shallow final fixture position.

The manufacturing tolerance of the metal sleeves and of the osteotomy drills may need to be included into the consideration of the errors. As an example, the company Blue Sky Bio (Blue Sky Bio, LLC, USA) [23], with as much as 75 different diameters of metal sleeves gives a manufacturing tolerance of its products of ± 50 μm. The clearance value should be estimated taking into account the expected manufacturing tolerances.

Finally, the abrasion of the sleeve, which is usually made of aluminium, caused during the drilling process, needs to be taken into account, especially for longer implants, where the drill engages the sleeve for a longer time. Horwitz et al. [24] found in their in vitro study that multiple uses of the drill and sleeve reduced the accuracy of the system. Cassetta et al. [12] using a modification on the External Hex Safe® (Materialise Dental, Leuven, Belgium) system (Group A) in order to minimise tolerance and reduce friction and damaging of the sleeve by the drill motion showed statistically improved accuracy in a retrospective clinical study. This could be interpreted as the best practice being the use of sleeves for a single time and the drills for the times recommended by the manufacturer. The abrasion of the components due to usage is not incorporated in our study.

## Conclusions

The results of this study compute part of the expected maximum differences in the final clinical implant position when a CAD/CAM surgical guide is used. Given this data, the implant surgeon, with an in-office 3d printer, has the equations and the tables to acknowledge the magnitude of the probable errors and to decide the best combination of the sleeve/drill parameters in order to minimise them. Practically, the implantologist can readily decide about the dimensions of the metal sleeve, the clearance between the osteotomy bur and the sleeve and the design of the guide in relation with the distance of the lip of the sleeve to the implant neck (offset), in order to find the best combination based on the available sleeves in the free market, the osteotomy burs he/she already possesses and the clinical situation in hand.

## Abbreviations

3d: Three dimensional
CAD: Computer-aided design
CAM: Computer-aided manufacturing
CBCT: Cone beam computed tomography
CI: Confidence interval
CT: Computed tomography
Dicom: Digital imaging and communications in medicine
FDM: Fused deposition modelling
GIS: Guided implant surgery
SLA: Stereolithography apparatus
STL: Surface tessellation language/stereolithography
